# Phonological characteristics of novel gesture production in children with developmental language disorder: Longitudinal findings

**DOI:** 10.1017/s0142716421000540

**Published:** 2021-12-15

**Authors:** Laiah Factor, Lisa Goffman

**Affiliations:** Callier Center for Communication Disorders, School of Behavioral and Brain Sciences, The University of Texas at Dallas, Richardson, TX, USA

**Keywords:** Gesture, DLD, SLI, Phonology, Motor Skill

## Abstract

Children with developmental language disorder (DLD; aka specific language impairment) are characterized based on deficits in language, especially morphosyntax, in the absence of other explanatory conditions. However, deficits in speech production, as well as fine and gross motor skill, have also been observed, implicating both the linguistic and motor systems. Situated at the intersection of these domains, and providing insight into both, is manual gesture. In the current work, we asked whether children with DLD showed phonological deficits in the production of novel gestures and whether gesture production at 4 years of age is related to language and motor outcomes two years later. Twenty-eight children (14 with DLD) participated in a two-year longitudinal novel gesture production study. At the first and final time points, language and fine motor skills were measured and gestures were analyzed for phonological feature accuracy, including handshape, path, and orientation. Results indicated that, while early deficits in phonological accuracy did not persist for children with DLD, all children struggled with orientation while handshape was the most accurate. Early handshape and orientation accuracy were also predictive of later language skill, but only for the children with DLD. Theoretical and clinical implications of these findings are discussed.

Developmental language disorder (DLD; also known as specific language impairment) affects approximately 7% of young children and is characterized as a deficit in language that is not explained by another disorder, such as hearing impairment, intellectual impairment, or autism (Bishop et al., 2017; Leonard, 2014; Tomblin et al., 1997; Tomblin et al., 1996). In the current work, DLD and SLI are used synonymously because the children in the DLD group met the inclusionary criteria for both DLD and SLI (see the method section below). Research concerning children with DLD has classically focused on language processing and production, particularly morphosyntax (Leonard, 2014), but phonology (e.g., Alt & Plante, 2006; Benham et al., 2018; Dollaghan & Campbell, 1998; Gray, 2005) and semantics (e.g., Kan & Windsor, 2010; McGregor et al. 2002; 2013b) have also been implicated. It has become apparent that children with DLD also show extra-linguistic deficits, such as in working memory (e.g., Archibald & Gathercole, 2006; Jackson et al., 2020; Lum et al., 2012) and motor skill (e.g., Brumbach & Goffman, 2014; Hill, 2001; Sanjeevan et al., 2015; Vuolo et al., 2017). Such crossdomain impairments suggest that the underlying mechanisms associated with DLD may not be specific to language.

The central issue addressed in the present work is whether the deficits attested in children with DLD are specific to spoken language, or if they manifest in, and can be explored through other modalities—in the present case, manual gesture. Gesture, like speech, is tightly linked to language (McNeill, 1985), incorporating meaning and form via motor implementation. However, unlike speech, gestures are often iconic and thus not arbitrarily linked to their meaning (McNeill, 1992). While gestures often show transparency in form-to-meaning mapping, their alignment to spoken and signed language (Goldin-Meadow & Brentari, 2017) affords the analysis of the conceptual and motor implementation components of phonological form.

Gestures are defined as abstract actions that do not physically change the environment, but influence behavior and cognition via communication (Goldin-Meadow, 2015; McNeill, 1985; 1992). In joining meaning to form, gestures can be assessed for both their conceptual and phonological components. As in speech, where constituent phonological features such as voicing or place of articulation can be subjected to fine-grained analysis, gesture can be broken down by phonological features, such as handshape, path, location, and orientation (Brentari, 1998; Brentari et al., 2017; Cheek et al., 2001; Liddell & Johnson, 1989; Pettenati & Stefanini, 2010; Sandler, 1989). In iconic (i.e., representational) gesture, handshape often reflects object characteristics, thus serving a noun-like function. The path of the hands often expresses functional information (Capone & McGregor, 2005; McNeill, 1985), including the trajectory and manner of the movement, capturing the verb-like aspects of gesture (e.g., Kita & Özyürek, 2003; Mumford & Kita, 2014; see also Goldin-Meadow et al., 1996). Location of the hands in reference to the body and the orientation of the hands to each other provide additional spatial information about the referent (see Brentari, 1998 for further discussion of orientation in sign). The fine-grained analysis of these phonological features in iconic gestures is the focus of the current work. The integration of meaning and form, as in spoken and signed language, is a central characteristic of gesture. That is, gesture provides a window into the intersection of language and motor systems in both typical and atypical development.

## Spoken word production in DLD

Many researchers have shown that there are form deficits in children with DLD that affect both morphosyntax (Goffman & Leonard, 2000; Leonard, 2014; Redmond & Rice, 2001; Rice & Wexler, 1996) and phonology (Benham et al., 2018; Goffman, 1999; Jackson et al., 2019). Both children and adults with DLD have difficulty producing novel word forms, likely driven in part by deficits in phonological sequencing (Benham et al., 2018; Heisler et al., 2010) and in encoding (e.g., Gathercole, 2006; Leonard et al., 2019; McGregor et al., 2013a; 2020). One of the most robust findings within the DLD literature is that, during the preschool and school-aged years, these children show deficits in nonword repetition tasks, with accuracy decreasing as word length increases (Deevy et al., 2010; Dollaghan & Campbell, 1998; Ellis Weismer et al., 2000; Graf Estes et al., 2007; Jackson et al., 2019). Preschool-aged children with DLD frequently have co-occurring speech sound deficits, as indicated by impaired performance on standardized articulation tests (Alt & Suddarth, 2012; Leonard, 2014; Shriberg et al., 1999; Vuolo & Goffman, 2018). While articulation deficits may diminish in the school years (Shriberg et al.,1994), it appears that deficits in encoding new word forms (McGregor et al., 2013a; 2020) and in phonological sequencing (Benham & Goffman, 2021) persist into the school years and beyond. In the present work, we ask whether these same deficits that are documented in speech production are also observed in the mapping of manual phonological form to novel gestures and whether these deficits continue from preschool into the early school years.

## Gesture production in children with DLD

While gesture appears to facilitate communication and lexical acquisition in children with DLD (e.g., Ellis Weismer & Hesketh, 1993; Evans et al., 2001; Mainela-Arnold et al., 2014; Vogt & Kauschke, 2017), there is some indication that, as in spoken words, there may be deficits in phonological form (Hill et al., 1998; Wray et al., 2016; 2017; cf. Botting et al., 2010). In studies of elicited and imitated single gesture production, children with DLD and typical development (TD) were asked to produce iconic gestures representing familiar objects (Botting et al., 2010; Wray et al., 2016) and/or actions (Hill et al., 1998; Wray et al., 2017). School-aged children with DLD were less accurate in their gesture productions (Hill et al., 1998; Wray et al., 2016; 2017; cf. Botting et al., 2010). For example, Wray and her colleagues reported decrements in gesture form accuracy in children with DLD when gestures were rated for overall accuracy on a 5-point Likert scale (Wray et al., 2016) and when gesture productions were scored based on a combination of form components (the hands’ shape, movement, and location; Wray et al., 2017). In addition, Hill and colleagues (1998) identified form errors associated with hand orientation and positioning in relation to the body in the elicited and imitated gestures of children with DLD. This pattern of error type suggests that the form deficits observed in the gestures of children with DLD may stem from phonological feature errors. However, the form errors reported in previous studies were analyzed as a motor rather than a linguistic skill; phonological features of sign language have generally not been considered. For example, the gesture analysis framework employed by Hill and her colleagues (1998) was derived from studies of adult apraxia. This analytic framework assumes a neuromotor, as opposed to phonological, source of the gesture errors produced by the children with DLD. Only a subset of the error types reported by Hill and her colleagues (1998) can be linked to deficits in phonological form—for example, the “external configuration” error, in which the child did not accurately represent the distance between the hand and the body, can be interpreted as a location error, but could also reflect a motor-based deficit. Other error types were judged as apraxic errors such as clumsiness or delayed imitation.

Wray and her colleagues (2016; 2017) report general form assessments, but do not apply a fine-grained analysis of individual phonological features. For example, Wray and her colleagues (2017) developed a holistic coding of accuracy that incorporated the phonological features of shape, path, and location, but did not differentiate their contributions. Their finding that children with DLD produced gestures less accurately than their TD peers suggests that form deficits may be phonological in nature. However, global measures of accuracy do not reveal the nature of the phonological deficit. It is also of note that Wray and her colleagues (2017) report a fine motor deficit in the children with DLD, leaving open the possibility that errors in gesture form may be tied to the motor system.

Gesture production, like speech, is phonological but also requires fine motor skill. Children with DLD have documented deficits in gross and fine motor skill, as evidenced by relatively weak performance on standardized tests compared to TD peers (Brumbach & Goffman 2014; Hill, 2001; Sanjeevan & Mainela-Arnold, 2019; Sanjeevan et al., 2015; Zelaznik & Goffman, 2010). However, more specific components of motor skill may be implicated, with both preschool- and school-aged children with DLD showing sequencing and coordination deficits in the manual domain on such tasks as bimanual clapping (Vuolo et al., 2017), serial reaction time tasks (Hsu & Bishop, 2014; Lum et al., 2014; Tomblin et al., 2007), and sequential handshape imitation (Marton, 2009; Wray et al., 2017). These findings, when considered in conjunction with the body of work showing motor implementation deficits in children with DLD, suggest that phonological errors in the speech and gestures of children with DLD may arise from a specific motor implementation impairment connected to sequenced or coordinated movement across multiple effectors (Vuolo et al., 2017).

## Current study

In the current work, we assessed the longitudinal progression of form aspects of gesture learning from preschool into the early school years. We also indexed fine motor and language development across this same developmental time period. A major objective was to determine the presence and persistence of early observed form-based deficits in the gestural domain.

The first theme of this work addressed the presence and developmental time course of phonological deficits in gesture production in young children with DLD. Children with DLD and TD participated in a three-time point (two year) longitudinal study beginning at age 4 to 5 years and continuing until 6 to 7 years of age. Children were tested annually in a task in which they were taught novel gestures in a word learning paradigm (described below). We asked whether the documented deficits in gesture production in young children with DLD (e.g., Hill et al., 1998; Wray et al., 2017) can be described phonologically and whether they persist into the early school years, mirroring phonological deficits in spoken language (e.g., Benham & Goffman, 2021; McGregor et al., 2013a). The presence of phonological deficits in the novel gesture productions of children with DLD would bolster claims of cross-modal influences that are not specifically tied to speech production. Conversely, if we observe no evidence of phonological deficits in gesture production in children with DLD, then, consistent with findings reported by Botting and colleagues (2010), we can infer that errors are driven by more general motor deficits (Hill et al., 1998; Wray et al., 2016; 2017). We are particularly interested in the developmental time course of phonological deficits in gesture production, since it is documented that form deficits in spoken language persist into later childhood (Benham & Goffman, 2021) and adulthood (McGregor et al., 2013a; 2020). Persistence of deficits in phonological aspects of gesture production from the preschool into the school years would provide evidence of a protracted cross-modal phonological impairment associated with DLD. However, it may be that phonological deficits are not fully cross-modal, with deficits in gesture production resolving over time.

A second theme centered on the phonological features of handshape, path, and orientation. While gesture and speech are tightly aligned within the linguistic system, the phonology of gesture differs from that of speech in that iconic gesture form is non-arbitrarily linked to its meaning (e.g., two hands in fists moving back and forth synchronously represent a swing or the act of swinging). The phonological features of handshape (tied to nouns) and path (tied to verbs) may be especially iconic (e.g., Capone & McGregor, 2005). We asked whether specific phonological features, including handshape, path, and orientation, showed deficits that extended into the school years.

Gestures, like speech, are of particular interest in typical and atypical development because they are thought to relate closely to both language and fine motor performance. The final theme addressed in this work is the relationship of both language and fine motor skill with gesture production. We asked whether early phonological feature accuracy in gesture was predictive of later language and/or fine motor outcomes. Children with DLD are defined by their language deficit, yet gross and fine motor skill are also frequently implicated in the disorder (Bishop et al., 2017; Vuolo et al., 2017). Gesture is, by definition, both motoric and linguistic. Thus, we asked whether early phonological accuracy in gesture production predicted either language or fine motor outcomes two years later.

## Method

### Participants

The children in this study participated in a series of experiments across a three-time point (two year) span focused on the relationship between language and action in children with DLD. Because we were interested in phonological aspects of gesture when children are 4 to 5 years old and the relationship to outcomes two years later, the current study concerns the first and final time points. Twenty-eight children (14 with DLD and 14 with TD) are included in the current analyses and represent a longitudinal subset of a larger time point one sample (*n* = 55, 34 with DLD and 21 with TD). These 28 children were the only children from the larger time point one sample to return at time point three to complete the novel gesture production task (described below) as well as the language and motor outcome measures. Independent sample *t*-tests confirmed that the 28 children who comprised the longitudinal sample did not significantly differ from the full time point one sample in performance on the standardized measures of language, cognition, and motor skill. The presence or severity of language impairment did not determine continued participation in the longitudinal study (Table 1).

The 14 children with DLD in the longitudinal cohort had a mean age of 4 years, 10 months (SD of 0;5 months; 6 females; 2 left-handed) at the study outset, as did the 14 children who were TD (M age of 4;10; SD of 0;5 months; 7 females; 2 left-handed). Upon return at time point three, the children with DLD had a mean age of 7 years, 7 months (SD of 0;6 months), and those with TD had a mean age of 7 years, 4 months (SD of 0;5 months). The children with DLD were recruited from a clinical research program for children with DLD at Purdue University. These children all received intervention during this clinical program that focused on literacy and vocabulary, not word form or gesture. Other aspects of therapy history are unknown. The children with TD were also recruited from the greater West Lafayette, Indiana area. They did not receive any speech or language interventions, and no history of a speech-language disorder was reported by their parents/legal guardians. Approval from the Institutional Review Board of Purdue University was obtained prior to the start of the study; the parents or legal guardians of the children provided written informed consent, and the children provided both written and verbal assent that was presented using developmentally appropriate language. The University of Texas at Dallas Institutional Review Board approved the data analyses included in the present study.

#### Inclusionary criteria

At study entry at time point one, all of the children performed within the typical range in nonverbal intelligence as measured by the Columbia Mental Maturity Scale—Third Edition (CMMS-3; Burgemeister et al., 1972). All of the children also passed a pure tone hearing screening (pure tones presented bilaterally at 20 dB HL at 500, 1000, 2000, and 4000 Hz), as well as a structural oral motor screener using the Robbins and Klee (1987) protocol. Finally, all children were rated as having minimal-to-no symptoms of autism spectrum disorder on the Childhood Autism Rating Scale— Second Edition (Schopler et al., 2010), and no history of neurological pathologies (e.g., seizures and head injuries) was reported by the parents.

#### DLD criteria for time point one

As is required for the diagnosis, the children included in the DLD group showed impairments in language abilities in accordance with the exclusionary criteria specified in Leonard (2014). A child was considered to have a language impairment if they received a standard score of 87 or below on the Structured Photographic Expressive Language Test—Preschool 2 (SPELT-P2; Dawson, et al., 2005), as this cutoff has been shown to be a sensitive (96%) and specific (95%) measure of DLD status (Greenslade et al., 2009). Children with DLD all participated in a summer clinical research program for which the SPELT-P2 served as the diagnostic measure. For the TD children, who did not participate in the summer program, the SPELT-3 (Dawson et al., 2003) was implemented. A standard score of 85 or below was used as the cutoff point. This cut-off point has been found to have 71.9% sensitivity and 100% specificity for detecting DLD according to Perona and colleagues (2005). The lowest standard score for the TD group was 90.

DLD status was further confirmed by a finite verb morphology composite score (comprised of percent correct production of regular past –*ed*, third-person singular –*s*, and copula and auxiliary forms of is, *are*, and *am*) that fell greater than 1.25 standard deviations below the mean based on normative data from the local West Lafayette, Indiana area (Goffman & Leonard, 2000; Leonard et al., 1999). Notably, the children with TD obtained typical finite verb morphology composite scores.

These results, as well as performance on the Bankson-Bernthal Test of Phonology (BBTOP; Bankson & Bernthal, 1990), are shown in Table 1. As is permitted for a diagnosis of DLD, performance on the BBTOP was free to vary (Leonard, 2014). The performance of the TD children on all measures was within expected levels.

#### Longitudinal measures

Language and motor ability were tracked across time points one and three for all of the children. At time point three, language ability was assessed using the Clinical Evaluation of Language Fundamentals—4^th^ Edition (CELF-4; Semel et al., 2003). This battery consists of four subtests: Concepts and Following Directions, Word Structure, Formulated Sentences, and Recalling Sentences. Scaled scores from each of the subtests were used to calculate a Core Language Score. Performance on the CELF-4 at time point three was free to vary; it was the initial diagnosis at time point one that was used to classify children with DLD.

Fine and gross motor skills were assessed using the Movement Assessment Battery for Children—2^nd^ Edition (MABC-2; Henderson et al., 2007). At time point one, children completed the 3- to 6-year-old age band, and at time point three, the 7- to 10-year-old age band. There are three areas assessed: Manual Dexterity, Aiming and Catching, and Balance. Standard scores from each of the subtests were used to calculate a Subtest Score and a Total Test Score. These scores were free to vary at both time points one and three as performance on the MABC-2 is not a part of the inclusionary-exclusionary criteria, and motor deficits are often identified in children with DLD (e.g., Hill, 2001; Vuolo et al., 2017).

**Figure 1.**
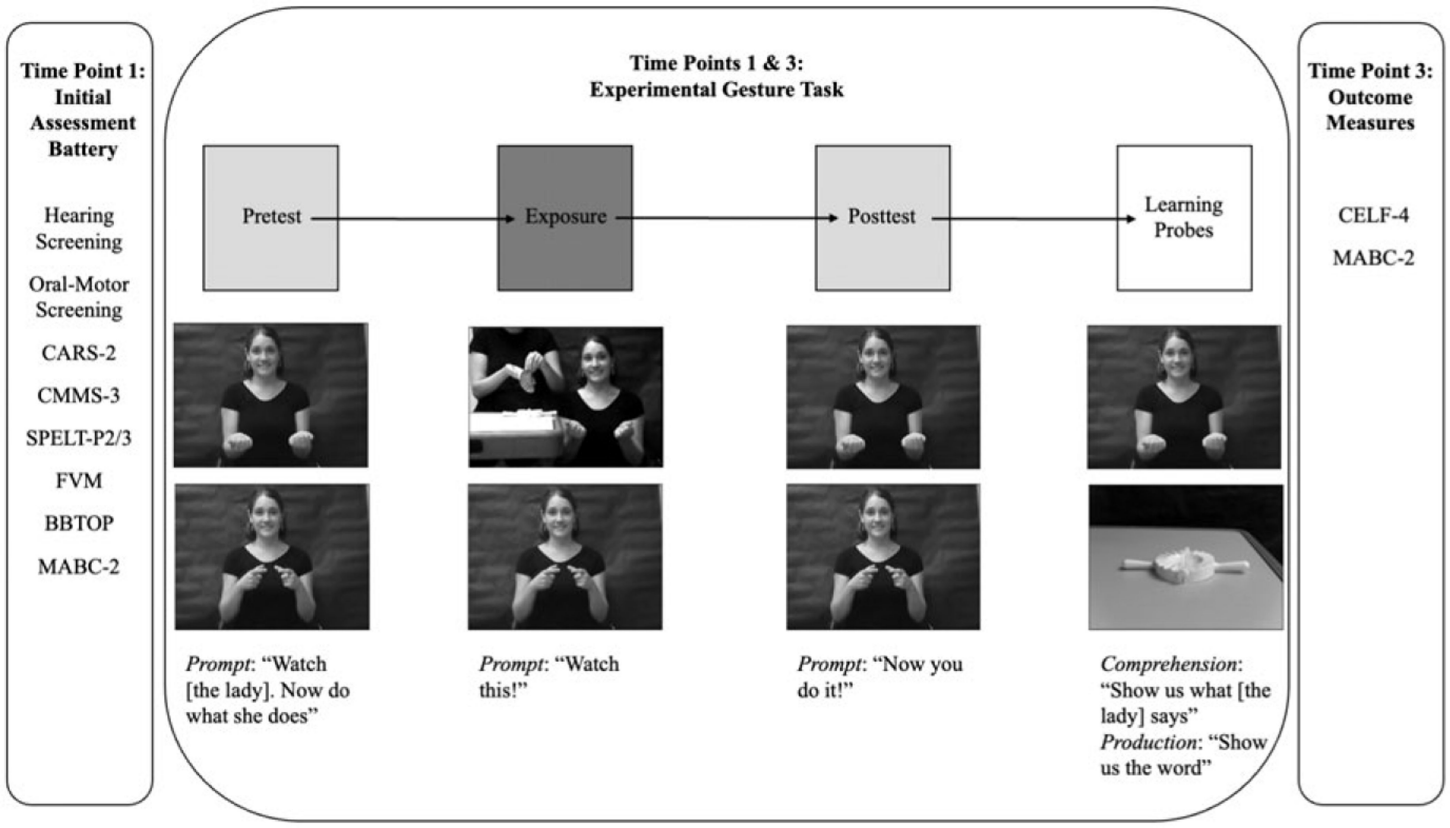
Experimental Protocol and Gesture Stimuli Presentation Schematic with Stimuli Exemplars and Prompts. CARS-2: Childhood Autism Rating Scale-2; CMMS-3: Columbia Mental Maturity Scale-3; SPELT-P2/SPELT-3: Structured Photographic Expressive Language Test-Preschool 2 or Structured Photographic Expressive Language Test-Preschool 3; FVM: Finite Verb Morphology; BBTOP: Bankson-Bernthal Test of Phonology; MABC-2: Movement Assessment Battery for Children-2; CELF-4: Clinical Evaluation of Language Fundamentals-4.

To summarize, at time point one, all of the children participated in a battery of assessments as shown in Figure 1. When the children returned at time point three, their language and motor skills were once again assessed now as outcome measures. At both time points, the children completed the experimental gesture task which is described in the next section.

### Experimental stimuli & procedure

The children were presented with four novel gestures and, in some cases, corresponding novel objects (unusual objects found at kitchen supply stores). The novel gestures were constrained to a set of four in this learning study because multiple productions (14 per gesture) were elicited and it was necessary to maintain a manageable number of productions for preschool-aged children. The novel gestures were constructed in accordance with American Sign Language phonotactic rules regarding handshape, path, and orientation (Brentari, 1998; Liddell & Johnson, 1989; Sandler, 1989) and were designed to be iconic, reflecting the attributes and affordances of the paired novel object (Capone & McGregor, 2005). The handshapes used in the novel gestures were drawn from those that are early developing in sign language (e.g., Boyes Braem, 1990; Marentette & Mayberry, 2000). The paths of the novel gestures were also designed to be simple single syllable movements as opposed to more complex multi-directional or repeated movements (e.g., an analogous speech production would be /ba/ versus /baba/ or /bapεf/). Table 2 shows pictures and descriptions of the novel gestures, as well as pictures of the novel objects. The study protocol, stimuli, and de-identified raw data can be accessed at https://osf.io/fr64e/.

Each novel gesture was recorded separately with and without the corresponding novel object referent in view. During the presentation of the stimuli, half of the novel gestures appeared with their referent and half appeared without their referent. The current work focuses on the phonological, rather than lexical, component of gesture production, and, in an analysis of the referent manipulation within the full time point one sample that is in preparation, no effects of referent were observed (*p* = 108); therefore, referential status was counterbalanced, but not analyzed. The videos were presented on a computer monitor display during data collection in a child friendly manner by a woman wearing a black shirt and seated against a dark gray background.

The children were seated in a child-sized chair at a table approximately 51cm in height and told that they were going to learn an alien language in which people talk with their hands. The children were video-recorded using a Panasonic HDC-HS700 camera for later phonological coding. Hand motions were also recorded, though not analyzed here.

The novel gestures were yoked into pairs and split into two blocks. Order of the novel gestures within a block was quasi-random (a gesture was never presented more than twice in a row). Each block was divided into three phases (Heisler et al., 2010; see Figure 1 for a schematic of the stimuli presentation across experimental phases):

#### Pretest Phase:

The child was presented with a video for two novel gestures (no object referents were included) and was instructed to watch and then imitate the gesture (7 times each).

#### Exposure Phase:

The child was passively exposed to the same two novel gestures; one of the gestures was presented without a referent, and the other was presented with its referent.

#### Posttest Phase:

Same as pretest.

Following the posttest phase of each block, comprehension and production probes were administered in a fixed order (comprehension then production):

#### Comprehension Probe:

The four novel objects were presented to the child. Two of the novel objects corresponded to the novel gestures presented during the experimental block, while the other two objects (foils) had not been shown to the child during the experimental block. The child was shown a video-recording of the woman producing one of the novel gestures that the child had been exposed to during the experimental block, and the child was instructed to find the object that corresponded to the gesture. The child’s response was binarily coded as correct (1 point) or incorrect (0 points).

#### Production Probe:

The child was presented with a picture of one of the novel objects and was asked to produce the corresponding gesture. The coding of the child’s response is described in the next section.

### Behavioral data analysis

#### Gesture coding

Gestures were analyzed for phonological accuracy in the pre- and posttest phases, as well as in the production probe. Gesture productions were coded for accuracy based on the phonological features of handshape, path, and orientation. Each phonological feature was coded on a three-point scale: two points were assigned if the feature was produced completely accurately (e.g., target handshape: closed fist/a-hand, child production: closed fist/a-hand), one point if aspects of the target feature were present in the child’s production but some errors were made (e.g., target handshape: closed fist/ a-hand, child production: cupped hand; in this example, the child has demonstrated partial knowledge of the target by bending their fingers), and zero points if the feature was completely inaccurate (e.g., target handshape: closed fist/a-hand, child production: flat hand). A maximum of six points per gesture production were possible (i.e., two points for handshape, two points for path, and two points for orientation). All pretest, posttest, and production probe productions were coded for phonological feature accuracy at the time points one and three; interrater reliability was established for time point one with 87.1% agreement and time point three with 90.2% agreement between the first author and two trained research assistants.

#### Analyses

Descriptive statistics are included to establish the linguistic and motor developmental profiles of the DLD and TD groups at time points one and three. Performance on the language measures was compared at time point one using an independent *t*-test while a one-way analysis of variance (ANOVA) was used at time point three to compare between-group performance on the composite language and subtest scores of the CELF-4. Separate analyses were used because, as appropriate for age, two different standardized language measures were administered across time points (the SPELT-P2/3 at time point one and the CELF-4 at time point three). Because the same standardized battery was used to index motor ability at each time point, a 2×3 (language group x MABC-2 subtest) mixed ANOVA with repeated measures was conducted to compare between-group (TD or DLD) performance (dependent variable) on the three subtests of the MABC-2 (within-subjects variable) at the first and final time points.

The children’s performance on the comprehension and production learning probes was also analyzed. Two 2×2 mixed ANOVAs were used. In each analysis, the effect of language group membership (between-subjects variable: TD or DLD) and time (within-subjects variable) on comprehension and production probe performance (percent correct), respectively, was assessed.

##### Phonological deficits over time.

The first and second themes addressed a) whether phonological deficits in the production of gesture were found when children were preschool-aged and persisted over time, and b) whether phonological feature accuracy differed within and across time points. These themes were addressed with a 2×3×2 (language group × phonological feature × practice) mixed ANOVA with repeated measures. This analysis assessed phonological accuracy (dependent variable) between language groups (TD or DLD), as well as across phonological features (handshape, path, and orientation) and practice (pretest and posttest) as a function of time (time points 1 and 3). Pairwise analyses were controlled for Type I error using a Bonferroni correction.

##### Phonological accuracy in relation to language and fine motor outcomes.

The final theme of this work assessed the longitudinal relationship between phonological accuracy in novel gesture production at time point one and language and fine motor outcome measures at time point three. This was done using a series of hierarchical regressions for each language group. The average time point one accuracy scores for each of the phonological features (handshape, path, and orientation), collapsed across pretest and posttest, were entered as predictor variables. The CELF-4 Recalling Sentences subtest scores at time point three were entered as the language outcome variable because this task has been found to be both sensitive and specific to language impairment status (Archibald & Joanisse, 2009; Conti-Ramsden et al., 2001). Recalling sentences tasks have also been shown to specifically reflect language skill, and not short-term memory or processing speed, in preschool-aged children with and without DLD (Klem et al., 2015; Plym et al., 2021). Likewise, fine motor skill intuitively is aligned with the production of gesture, and children with DLD have previously been reported to demonstrate deficits in fine motor ability, including manual sequencing and coordination (Marton, 2009; Sanjeevan & Mainela-Arnold, 2019; Vuolo et al., 2017; Wray et al., 2017). Therefore, the MABC-2 Manual Dexterity subtest scores were entered as the fine motor outcome variable.

Finally, because nonverbal ability may influence the production of gesture, we included an analysis of nonverbal scores. The time point one CMMS-3 scores were entered as a covariate in all of the hierarchical regressions. As noted in the inclusionary criteria section above, all of the children, regardless of language ability, scored within the typical range on the CMMS-3 with the lowest score being a 94 (Table 1). Nonetheless, to account for the potential influence of nonverbal ability, this covariate was included.

## Results

### Performance on the standardized language and motor measures

Language ability of the children with DLD was compared to their TD peers to assess performance at time point one and at time point three. Recall that children were assigned to the DLD group based on performance at time point one, and time point three was free to vary. Therefore, as expected, a between-language group comparison of the time point one language scores confirmed that the language ability of the TD and DLD groups was significantly different, *t*(26) = 8.78, *p* = .000 (TD: M = 109.07, SD = 11.63; DLD: M = 74.07, SD = 9.33). At time point three, the children with a diagnosis of DLD at age 4 to 5 years (time point one) still showed language scores significantly lower than the TD group as measured by both the CELF-4 core language score, *F*(1,24) = 16.50, *p* = .000, η_p_^2^ = 0.30 (TD: M = 108.79, SD = 12.46; DLD: M = 89.00, SD = 12.29) and the CELF-4 recalling sentences subtest score, *F*(1,26) = 8.10, *p* = .006, η_p_^2^ = 0.20 (TD: M = 10.42, SD = 3.48; DLD: M = 7.14, SD = 2.18); see Table 3 for all CELF-4 subtest between-group comparisons. However, it is important to note that at time point three, only three of the fourteen children in the DLD group had a CELF-4 core language score greater than one standard deviation below the mean. Six of the fourteen children with DLD scored greater than one standard deviation below the mean on the recalling sentences subtest. Based on this measure, language disorder did not persist for several of these children.

The three subtests of the MABC-2 (Manual Dexterity, Aiming and Catching, and Balance) were used to index the children’s fine and gross motor skills at the first and final time points. Overall, there was a main effect of language group, *F*(1,26) = 4.24, *p* = .05, η_p_^2^ = 0.14, with the TD group demonstrating higher motor subtest scores, but no main effect of time, *F*(1,26) = 1.70, *p* = .204, η_p_^2^ = 0.06. Additionally, there was a group by subtest interaction, *F*(2,52) = 3.52, *p* = .037, η_p_^2^ = 0.12. TD children scored higher on the Balance subtest (M = 11.82, SD = 2.64) than did the children with DLD (M = 8.96, SD = 3.56), *p* = .004. Though not statistically significant, the TD children also scored higher on the Manual Dexterity subtest (M = 10.25, SD = 2.27) in comparison to the children with DLD (M = 8.39, SD = 3.22), *p* = .056. The groups did not significantly differ on the Aiming and Catching subtest, *p* = .90. Table 4 shows the group comparison of means, standard deviations, and score ranges for the MABC-2 total test scores and subtests at each time point.

Although the group comparison of the Manual Dexterity subtest, which indexes fine motor skill, was not statistically significant, the DLD group was notably more variable in their fine motor performance than their TD peers. At time point one, all but one TD child scored within expected levels on the fine motor tasks that comprise the Manual Dexterity measure, while over a third of the children with DLD (5 out of 14) scored one standard deviation below the mean. When the children returned at time point three, all of the TD children scored within the typical range of the Manual Dexterity subtest, while 4 of the 14 children with DLD scored below one standard deviation of the mean.

### Performance on experimental tasks

#### Learning probes

Comprehension and production probes were administered following the posttest phase. All children performed at similar levels on both the comprehension probe, *F*(1,26) = 0.00, *p* = 1.00, η_p_^2^ = 0.00 (TD: M percent correct = 82%, SD = 39%; DLD: M percent correct = 82%, SD = 39%), and production probe,*F*(1,26) = 1.77, *p* = .195, η_p_^2^ = 0.06 (TD: M percent correct = 72%, SD = 26%; DLD: M percent correct = 66%, SD = 27%). There was a significant improvement in performance for all children on the comprehension probe, *F*(1, 26) = 14.60, *p* = .001, η_p_^2^ = 0.36, as well as on the production probe from time point one to three, *F*(1,26) = 61.13, *p* = .000, η_p_^2^ = 0.70, as shown in Table 5. No interaction effects between group and time for either probe were found.

#### Phonological deficits over time

We began by asking a) whether phonological deficits in gesture production were observed at time point one and, if so, whether they persisted to time point three, and b) which, if any, phonological features showed group differences or relative weaknesses at each time point.

There was a main effect of language group: the children with TD demonstrated higher phonological accuracy (M = 1.62, SD = 0.28) compared to the children with DLD (M = 1.50, SD = 0.33), *F*(1,26) = 9.2, *p* = .005, η_p_^2^ = 0.26. There was also a main effect of time, *F*(1, 26) = 56.4, *p* = .000, η_p_^2^ = 0.68; all of the children significantly improved in phonological accuracy from time point one (M = 1.46, SD = 0.32) to time point three (M = 1.66, SD = 0.27). The children were not equally accurate across phonological features, *F*(2,52) = 34.0, *p* = .000, η_p_^2^ = 0.57. Handshape (M = 1.67, SD = 0.20) and path (M = 1.66, SD = 0.33) were both more accurate than orientation (M = 1.35, SD = 0.28), *p* = .000, but were similarly accurate to one another, *p* = 1.00. There was no main effect of short-term practice (pretest to posttest), *F*(1,26) = .004, *p* = .949, η_p_^2^ = .000.

**Figure 2.**
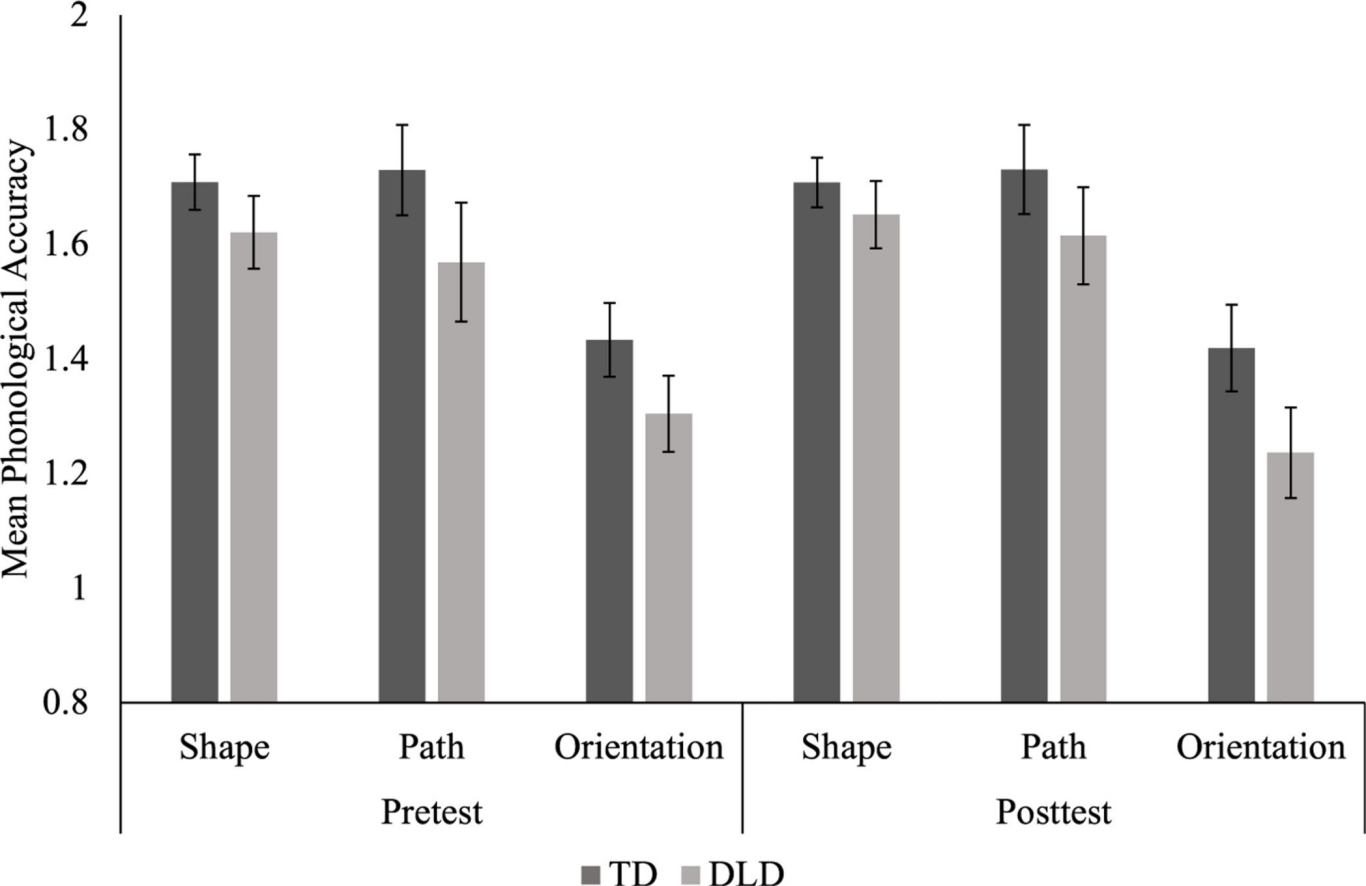
Mean Phonological Accuracy by Feature Across Pre- and Posttest and Between Groups with Standard Error. The maximum overall phonological accuracy score is six points. When accuracy scores are separated by phonological feature (shape, path, and orientation), a maximum of two points per feature is possible.

**Figure 3.**
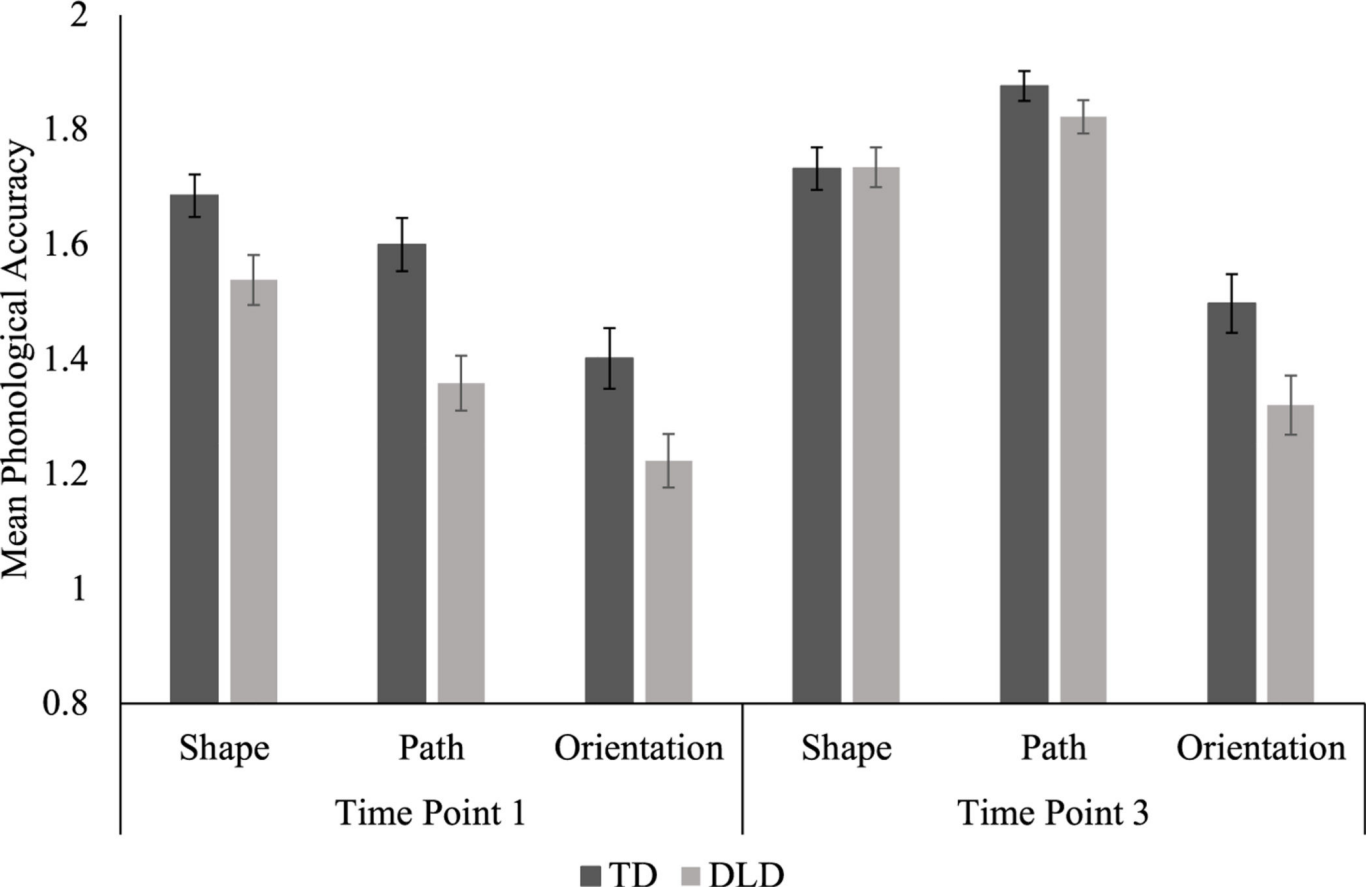
Mean Phonological Accuracy by Feature Across Time Points and Between Groups with Standard Error. The maximum overall phonological accuracy score is six points. When accuracy scores are separated by phonological feature (shape, path, and orientation), a maximum of two points per feature is possible.

There were two significant interactions: phonological feature accuracy by pretest-posttest, *F*(2,52) = 4.5, *p* = .015, η_p_^2^ = 0.15, and phonological feature accuracy by time point, *F*(2,52) = 5.1, *p* = .009, η_p_^2^ = 0.16. Both interactions reflect the relative weakness of orientation accuracy. Specifically, both before and after the exposure phase, orientation was significantly less accurate than handshape, *p* = .000, and path, *p* = .000. Orientation was also the only feature to not significantly improve from time point one to three, *p* = .09; both handshape, *p* = .003, and path, *p* = .000, demonstrated significant longitudinal gains; see Figures 2 and 3.

#### Phonological accuracy in relation to language and fine motor outcomes

We also examined the predictive relationship between preschool-aged gesture phonological feature accuracy and school-aged language and fine motor outcomes. Using a series of planned hierarchical regressions, each group’s time point one phonological feature accuracy scores averaged across pretest and posttest (due to the null main effect of short-term practice) were entered separately as the predictor variables while the time point three CELF-4 Recalling Sentences scores and the MABC-2 Manual Dexterity scores were entered as the outcome variables, respectively. Nonverbal ability at time point one, as indexed by the CMMS-3, was entered as a covariate for all regressions.

**Figure 4.**
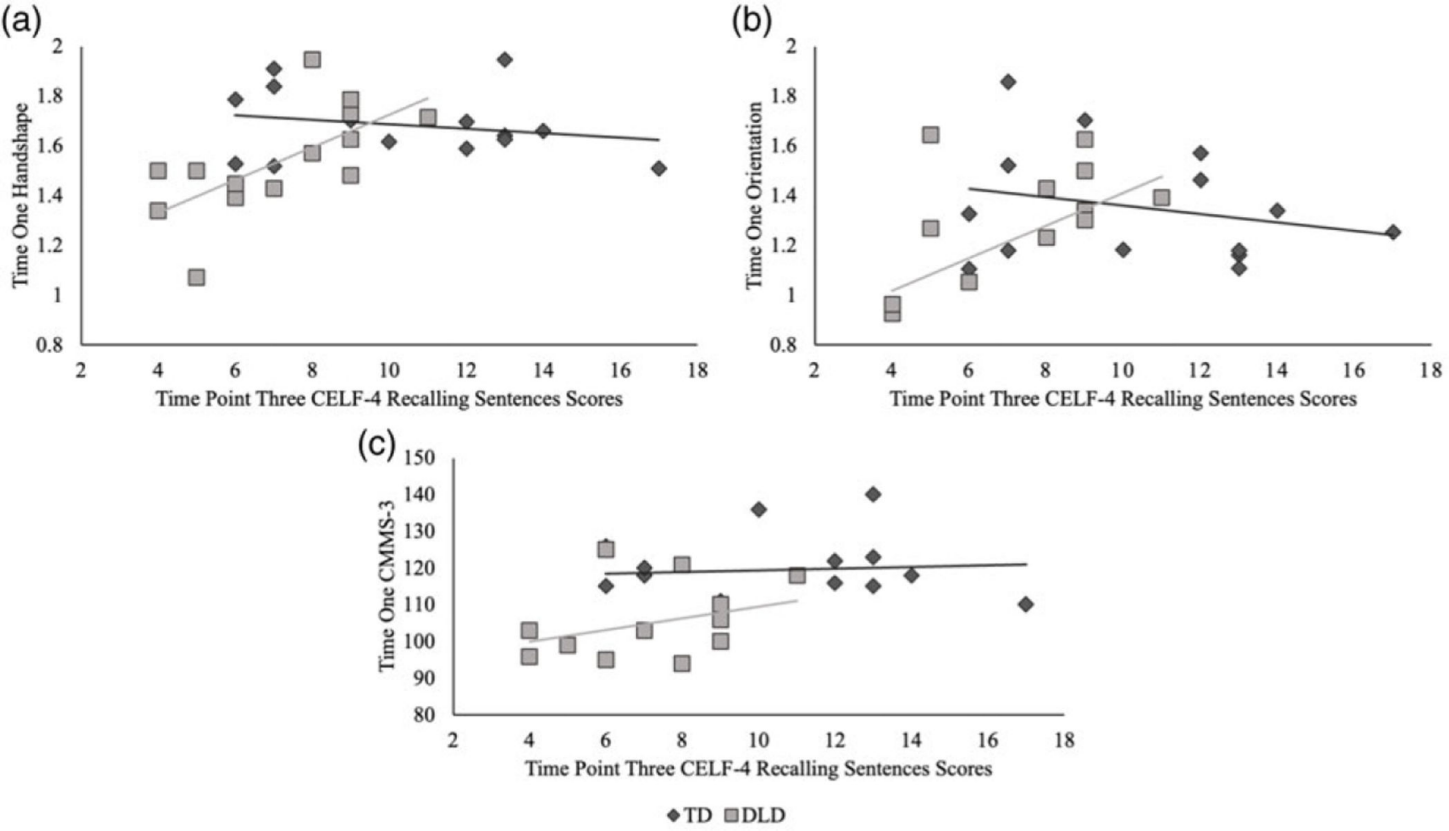
Time Point One Significant Predictors for Time Point Three Outcome CELF-4 Recalling Sentences Scores. a) Time point one mean handshape phonological accuracy; b) time point one mean orientation phonological accuracy; c) time point one nonverbal CMMS-3 scores.

For the children with DLD, the hierarchical regression analysis of time point three language ability demonstrated a combined significant effect across all three predictor variables (time point one handshape, path, and orientation accuracy) as well as the nonverbal covariate in the final model, *F*(4,9) = 6.02, *p* = .012, R^2^ = .72 (adjusted R^2^ = .61); see Table 6. An examination of the individual predictor variables indicated that time point one handshape accuracy was the largest significant predictor (ß = .608, *t* = 3.05, *p* = .014), followed by time point one orientation accuracy (ß = .450, t = 2.45, *p* = .037), and nonverbal ability (ß = .411, *t* = 2.28, *p* = .049). Path accuracy at time point one failed to reach significance at all model stages (*p* = .445). As shown in Table 6, time point one handshape accuracy was a significant predictor at every model stage and the covariate of nonverbal ability did not reach significance until the final model when time point one orientation was entered. It is also of note that the standardized ß coefficients for time one orientation accuracy and nonverbal ability are comparable, indicating that both variables contribute to the final model to a similar degree. However, there is no evidence of multicollinearity between time point one orientation accuracy (variance inflation factor = 1.11, tolerance = .90) and nonverbal ability (variance inflation factor = 1.08, tolerance = .93). Conversely, no significant relationship between phonological feature accuracy, nonverbal ability, and later language ability was found for the TD children, *F*(4,9) = 0.52, *p* = .721, R^2^ = .19 (adjusted R^2^ = .00), as shown in Figure 4.

**Figure 5.**
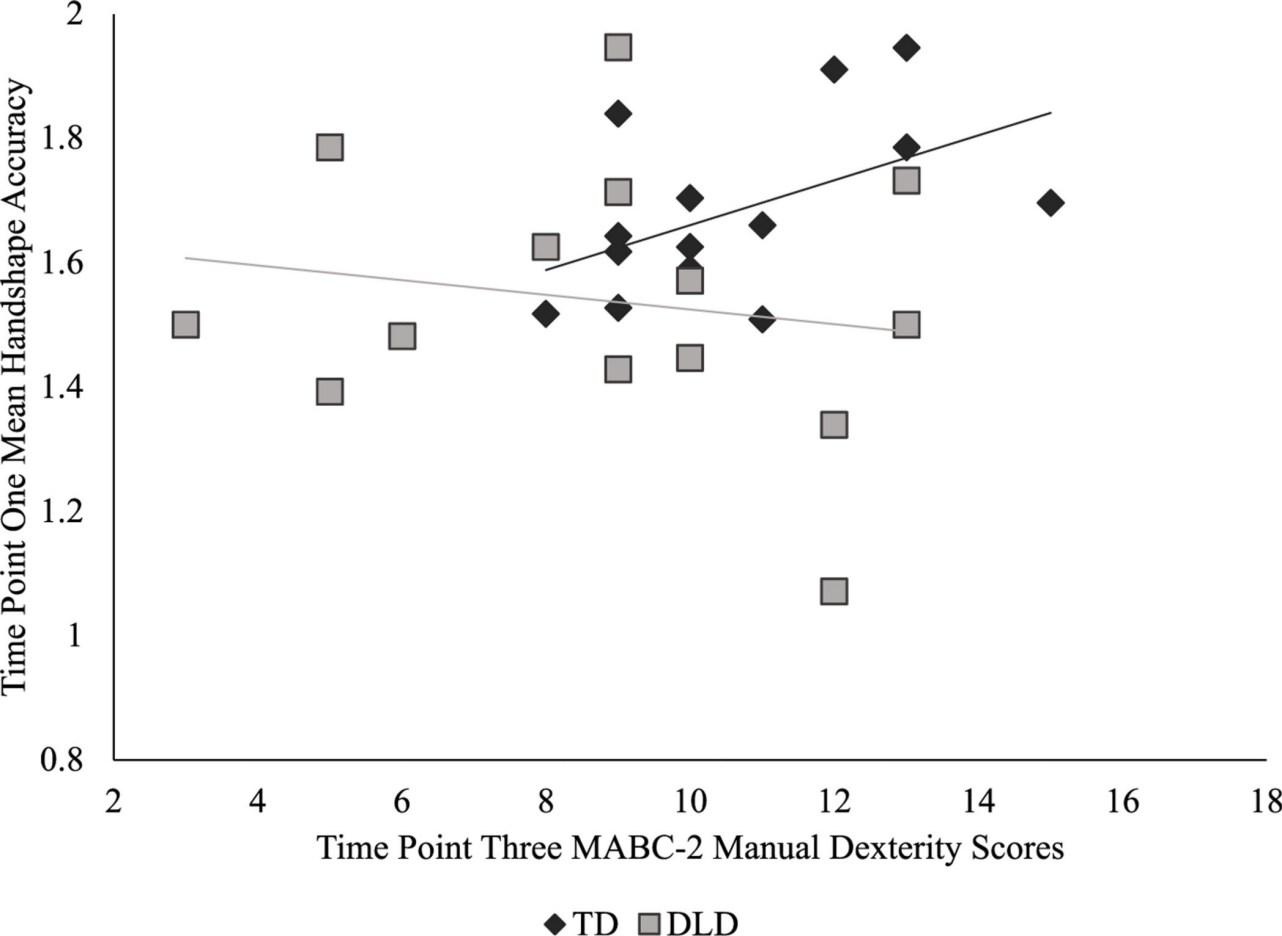
Time Point One Mean Handshape Phonological Accuracy and Time Point Three MABC-2 Manual Dexterity Scores.

Turning to the time point three MABC-2 Manual Dexterity scores, the hierarchical regression analyses failed to yield a significant predictive relationship between time point one phonological feature accuracy, nonverbal ability, and time point three fine motor skill for both children with DLD, *F*(4,9) = 0.83, *p* = .537, R^2^ = .27 (adjusted R^2^ = .00), and children with TD, *F*(4,9) = 1.70, *p* = .233, R^2^ = .43 (adjusted R^2^ = .18). As an example, Figure 5 displays the null relationship between time point one handshape accuracy and time point three MABC-2 Manual Dexterity scores for the TD and DLD groups; a similar pattern of results was found for path and orientation. Note that at time point three, four of the children with DLD demonstrated a fine motor impairment; however, this did not relate to earlier phonological gesture accuracy.

#### Summary of results

Analysis of the children’s phonological accuracy showed that, while children with DLD are generally less accurate than their TD peers, all of the children improved from time points one to three. The phonological deficits observed in the gestures of the children with DLD when they were preschool-aged did not persist. Further examination revealed differential degrees of accuracy between phonological features: handshape and path were more accurate than orientation; only orientation failed to improve in accuracy in response to practice and time. Finally, handshape accuracy, orientation accuracy, and nonverbal ability at time point one were found to be positively linked to later time point three language ability, but only for children with DLD. No longitudinal links between early phonological accuracy, nonverbal ability, and later fine motor ability were found.

## Discussion

In the current work, we explored whether and how phonological features in novel gesture production predict language and motor outcomes in children with DLD. Gestures, like speech, recruit both the linguistic and motor systems; however, unlike speech, the form-to-meaning mapping found in iconic gestures is transparent and non-arbitrary. This pattern of overlap and divergence in speech and gesture allowed us to explore three themes regarding the conceptual and motor systems’ contributions to gesture phonological production accuracy in children with TD and DLD, including developmental change over time.

### Theme 1: Phonological deficits over time

First, we asked whether there were deficits in the phonological accuracy of gestures in preschool-aged children with DLD, as well as whether these deficits persisted into the early school years. The presence and persistence of these phonological errors into the early school years would indicate that the form-based deficits documented in the production of novel spoken language cross to the manual modality. While children with DLD did demonstrate a phonological deficit in gesture, it appeared to resolve in the production of simple iconic gestures. All of the children, whether DLD or TD at study entry, demonstrated significant gains in overall phonological gesture accuracy between the preschool- and school-aged years, with the children in the DLD group converging on typical levels of accuracy by time point three.

The initially weak performance and positive developmental gains in phonological accuracy observed for children with DLD cannot be attributed to deficits in the conceptual encoding of the novel gestures. Analysis of the learning probes at each time point indicates that the form-based production deficits observed when the children with DLD were in preschool were not connected to their ability to receptively and expressively map the novel gestures to their referents. In fact, there were no group differences in the form-to-meaning mapping accuracy on the comprehension and production probes at either time point. This mirrors previous research showing that word form deficits are central in children (Benham et al., 2018) and adults with DLD (McGregor et al., 2013a; 2020). It appears that relative phonological weakness in the production of simple iconic gestures decreases over time for children with DLD and TD alike.

The convergence to typical levels in phonological gesture accuracy differs from the pattern observed in novel word form production in speech, which has shown deficits that persist into adulthood (e.g., McGregor et al., 2013a). This dissociation between gesture and spoken word form could be due in part to the nature of the form-to-meaning connection in speech versus gesture. While the meaning of an iconic gesture is inherent in its form, this same intrinsic relationship between meaning and form is not obligated in speech (though iconicity may be found in both speech and sign; for example, Goldin-Meadow & Brentari, 2017; Perniss & Vigliocco, 2014). As such, the link between the conceptual and phonological levels may lead to more phonologically accurate gesture production.

### Theme 2: Phonological feature accuracy

While the children’s overall phonological gesture accuracy increased across time points, the degree of improvement in individual phonological features was not equivalent. The phonological features of handshape and path were produced more accurately by all of the children in comparison to hand orientation. Orientation, unlike handshape and path, failed to show significant gains in accuracy in response to practice (pretest to posttest), as well as maturation (time points one to three). The unexpected persistence of weakness in orientation, independent of language ability, signals the presence of phonological markedness in iconic gestures; handshape, as well as path, may be more perceptually salient or semantically rich in comparison to orientation. Orientation may draw upon different conceptual constructs in comparison to handshape and path.

Other work has shown that specific features of gestures may influence learning. When paired with speech, handshape in iconic gesture is especially facilitative of novel spoken word learning in both typical toddlers (Capone & McGregor, 2005), as well as toddlers classified as late talkers (Capone Singleton & Anderson, 2020). It is hypothesized that this is because handshape, more so than path, emphasizes salient key conceptual features of the referent that disambiguate it from other possible referents. For example, the action of drinking from both a cup and a mug can be represented by lifting the hand to the lips; it is the handshape that differentiates between these two objects (a cupped hand versus a closed fist). In this way, handshape carries a high level of saliency because it adds specificity to the abstract action of gesturing (Goldin-Meadow, 2015), thus laying the concrete representational foundation that characterizes iconic gestures.

In contrast, hand orientation in gesture may be more conceptually abstract and more closely aligned to visuospatial processing and mental imagery (e.g., Frick et al., 2009; Levine et al., 2018; Shepard & Metzler, 1971), both of which undergo developmental changes during early childhood (e.g., Dukette & Stiles, 2001; Frick et al., 2013). When conveying the (dynamic) positioning of a referent in space via gesture, one is engaging in a form of mental imagery wherein visual, haptic, and proprioceptive knowledge of the affordances of one’s own body as well as an object’s affordances are drawn upon (e.g., Amorim et al., 2006; Barsalou, 2008). This sensorimotor knowledge is reflected in the orienting of the palms in reference to each other, the body, and the interlocutor.

While mental imagery and visuospatial processing were not directly measured in the current research, it is perhaps unsurprising that all of the children, and the children with DLD to a slightly greater degree, struggled with hand orientation. The production of novel iconic gestures presumably taps multiple representational levels—handshape and path reflect concrete conceptual elements of the referent and are tied to the word classes of nouns (handshape) and verbs (path; e.g., Capone & McGregor, 2005). Orientation in gesture does not have a direct word class analogue and often serves as a modifier by relaying additional information about the spatial positioning of the referent. For example, whether a cupped hand representing a glass is upright or tilted to palm down communicates two different states of the glass and its contents. The spatial positioning of the hand may have changed, but what did not change was the fact the handshape represented a glass.

When producing the novel gestures included in the current study, the children appeared to be sensitive to the phonological features that conveyed the majority of the key semantic information, leaving orientation and the spatial information it conveys to vary. In leaving orientation to vary, the children were still preserving the core of the gesture form—the shape of the object (the noun) and its movement/function (the verb). The visuospatial information conveyed by orientation was conceptually, and perhaps perceptually, peripheral to the core form-to-meaning link in this gestured word learning task. As such, the effect of practice and time on orientation accuracy was diminished. This, in conjunction with the ongoing development of mental imagery abilities, may have led to the persistent weakness in orientation that was observed for all of the children.

### Theme 3. Phonological accuracy in relation to language and fine motor outcomes

Finally, we assessed whether the production of handshape, path, and orientation during the preschool years related to later language and fine motor outcomes. Handshape and orientation accuracy, as well as nonverbal ability at time point one when the children were preschool-aged were positively predictive of later language ability, but only for the children with DLD. None of the phonological features nor nonverbal ability at time point one were linked to later language skill for the TD children. There were no relationships in either group between phonological feature accuracy or nonverbal ability during the preschool years and later fine motor development. These findings suggest that phonological factors, rather than fine motor ability (Hill et al., 1998; Wray et al., 2017), drive the form deficits observed in gesture production in children with DLD.

#### Gesture as a part of the language system

The connection between phonological gesture accuracy and language, rather than fine motor outcomes, aligns with unified system theories of gesture, language, and cognition (e.g., Goldin-Meadow, 2002; McNeill, 1992; 2005). These theories argue that gesture exists within an interconnected conceptual network. Within this network, gesture augments and combines with language to convey meaning. The longitudinal relationship between gesture phonology and language skill fits into this theoretical framework by reinforcing the shared conceptual underpinnings underlying gesture and language, as opposed to gesture functioning as a separate representational act (e.g., Krauss et al., 2000). Of course, phonological accuracy in gesture is derived in part from fine motor skill. However, the novel iconic gestures used in this study were simple productions that incorporated early developing handshapes and were produced in isolation. These gestures presumably required fewer motor demands than those that are embedded in a series of contiguous gestures or coproduced with speech. Gesture production accuracy has been linked to manual dexterity regardless of language impairment when multiple iconic gestures were concatenated or multiple effectors were recruited (e.g., including both the hands and the feet when producing a “ladder” gesture; Wray et al., 2017).

Because phonological accuracy was assessed over numerous gesture productions, the influence of fine motor ability may have decreased as the children practiced the novel gestures. It is possible that manual dexterity would be found to affect phonological accuracy during initial stages of novel gesture fast-mapping when cognitive demand is high. In the spoken domain, higher cognitive demand has been shown to increase articulation variability in children with DLD, whereas repetition decreased variability (Saletta et al., 2018). Repetition over time has also been shown to decrease spoken word form errors (e.g., Leonard et al., 2020). As such, the motor and linguistic systems differentially influence speech production at different stages of learning. While gesture phonology appears to be tightly linked to the linguistic system, the contribution of fine motor ability during initial stages of gesture production remains open to investigation.

#### Gesture phonology, nonverbal ability, and DLD

The finding that time point one gesture accuracy, specifically handshape and orientation accuracy, was tied to the later language ability of the children with DLD and not their TD peers may provide particular insight into the nature of DLD. If the phonological accuracy of gesture is simply a reflection of language skill, then it would be expected that it would be tied to language gains for all of the children; however, this was not the case. Both children with DLD and their TD peers demonstrated varying degrees of handshape and orientation accuracy at time point one, as well as a range of language performance scores at time point three (Figure 4), yet the longitudinal effect of handshape and orientation accuracy (to a lesser degree) on later language skill was only found for the children with DLD.

In one account of DLD, the Procedural Deficit Hypothesis (Ullman et al., 2020; Ullman & Pierpont, 2005), it is hypothesized that the declarative system is relatively spared and may act as a compensatory mechanism for an impaired procedural system. The spared declarative system is responsible for word learning in isolation, including the binding of form and meaning (e.g., Ullman & Pierpont, 2005). Handshape may tap into this preserved conceptual system more so than other phonological features. As discussed above, handshape plays a core role in disambiguating key distinguishing physical characteristics of a referent, thus capitalizing on conceptual knowledge and facilitating the connection between meaning and form. The present finding that handshape positively predicts later language skill, but only for children with DLD, supports that the conceptual system of children with DLD is preserved and provides a compensatory mechanism for language, thus representing a source of resiliency.

In addition to handshape, early orientation accuracy and nonverbal ability were also found to relate to improved language skill in the children with DLD when they were school-aged. As discussed above, orientation may be connected to more conceptually abstract mechanisms such as mental imagery and visuospatial processing. Both mental imagery and visuospatial processing represent cognitive mechanisms that support nonverbal problem solving and reasoning (i.e., fluid intelligence; Gillam et al., 2019). Indices of nonverbal reasoning have been linked to nonverbal hand position imitation tasks in preschool-aged children with DLD, but not their TD peers (Plym et al., 2021), a pattern that was found in the current study as well. It is possible that these tasks tap into a general nonverbal processing mechanism for children with DLD. While dynamic nonverbal visuospatial tasks have been identified as an area of weakness in DLD (e.g., Gillam et al. 2019; Savich, 1984; Ullman et al., 2020; Ullman & Pierpont, 2005), it is possible that the children with DLD who demonstrated better orientation accuracy or nonverbal ability in the current work were able to recruit an underlying nonverbal processing mechanism such as sustained visuospatial attention (e.g., Smolak et al., 2020) to support their impaired language system, allowing for greater language gains longitudinally. This interpretation of the positive longitudinal relationship between early orientation accuracy, nonverbal ability, and later language ability in children with DLD is speculative and requires further investigation.

The pattern of the regression analysis results for the children with DLD suggests that there are areas of underlying resiliency that may fulfill compensatory or facilitatory roles for language development within an impaired language system. The origin of this resiliency may lie in the conceptual system as suggested by the relationship between handshape accuracy and language, as well as domain-general mechanisms such as visuospatial processing. The origin and nature of this resiliency as well as how it may contribute to improved language ability in children with DLD require further investigation; however, these findings help to further characterize the nature of the cognitive mechanisms that give rise to the pattern of strengths and weaknesses associated with DLD.

A parallel relationship between early gesture accuracy or nonverbal ability and later language skill was not found for the TD children. This null result cannot be attributed to all of the TD children clustering around a similar language score when school-aged; children with TD demonstrated a range of language skills (Figure 4). Nonetheless, these children had typically developed language systems. Gesture has been shown to foreshadow linguistic development in typically developing toddlers (e.g., Iverson & Goldin-Meadow, 2005). However, by the preschool years, gesture assumes a supplementary role with language (signed or spoken) carrying the burden of communication; gesture may no longer provide direct insight into language development. Conversely, when language is impaired, the role of gesture as a primary communication facilitator persists (Evans et al., 2001; Lavelli & Majorano, 2016; Wray et al., 2017). In this way, gesture, specifically handshape and orientation, appears to continue to provide insight into the language systems of children with DLD, including perhaps serving as an indicator of sources of resiliency.

### Future directions, limitations, and conclusions

The connection between early phonological gesture accuracy and later language skill in DLD provides many possible avenues of further inquiry. Key is continuing to identify preserved underlying cognitive mechanisms, as these may serve as points of strength for children with DLD. It is possible that the connection between gesture and conceptual knowledge extends beyond a compensatory relationship and may offer a means to promoting improvements in language ability for children with DLD. Future work utilizing larger sample sizes than in the present work, as well as novel methodologies, should continue to investigate the gesture-language link in both imitated and spontaneous gesture.

Also requiring further investigation are the divergent cognitive mechanisms that underlie the phonological features of gesture. We have put forth one possible explanation as to why orientation is generally harder for children independent of language ability, as well as why it still may be related to language development in DLD; however, this phonological feature is understudied in both children and adults.

Likewise, the investigation of handshape and its tie to concepts and vocabulary acquisition holds promise for language impaired populations, but this line of inquiry is still in the early stages. The current study did not directly measure the general cognitive mechanisms that may relate to the different phonological features of gesture. To better understand the connections between gesture, language, and broader cognition, an important next step is to unpack the cognitive mechanisms that underlie gesture features.

Finally, while preschool-aged children with DLD were less phonologically accurate during novel gesture production than their TD peers, this deficit did not persist into the early school years. The DLD group’s gesture phonological accuracy ultimately converged to typical levels. We theorized that this resolution of the gesture phonological deficit was due to the role of iconicity in the form-to-meaning link. However, the novel gestures were designed to be simple in form and this simplicity cannot be ruled out as a contributing factor. Future work that incorporates more complex gestures designed to tax the language and motor systems is required to better understand the scope and duration of cross-modal phonological deficits in DLD.

In sum, we have shown that phonological deficits occur cross-modally in the gestures of preschool-aged children with DLD. However, these phonological deficits in gesture do not persist. Fine-grained analyses revealed differential levels of phonological accuracy within and across time points. Handshape and path were highly accurate, while orientation accuracy was comparatively lower and did not improve over time. Finally, we explored the longitudinal relations between early phonological accuracy and later language and fine motor skills. Early handshape accuracy, orientation accuracy, and nonverbal ability were found to be positively predicative of later language skill, but only for children with DLD. Taken together, this study has shown that the phonological systems of children with and without DLD can be explored cross-modally and that the phonological features of handshape and orientation provide insight into the underlying linguistic system. While it is important to not lose sight of gesture’s holistic nature and how this contributes to communication, more fine-grained analyses of gesture’s components may aid in furthering our understanding of gesture as a window to both language and cognition within both typical and atypical development.

## Figures and Tables

**Table 1.** Group comparisons, means, and standard deviations for standardized tests at time point one

		TD		DLD		
				
Measure		Mean *(SD)*	Range	Mean *(SD)*	Range	*p*-value^a^

**SPELT-P2/SPELT-3**	**Full Sample**	111.3 *(11.1)*	90–130	76.9 *(8.5)*	61–87	<.001

	**Longitudinal Sample**	109.1 *(.116)*	90–125	74.1 *(9.3)*	61–87	<.001

	***p*-value** ^b^	.58		.34		

**Finite Verb Morphology**	**Full Sample**	92% *(10%)*	61–100%	55% *(26%)*	16–97%	<.001

	**Longitudinal Sample**	90% *(12%)*	61–100%	47% *(22%)*	19–85%	<.001

	***p*-value**	.63			.24		

**BBTOP**	**Full Sample**	100.1 *(8.5)*	86–117	79.6 *(12.0)*	65–108	<.001

	**Longitudinal Sample**	99.0 *(10.0)*	86–117	75.1 *(10.4)*	65–90	<.001

	***p*-value**	.74			.37		

**Nonverbal Intelligence**	**Full Sample**	116.7 *(9.1)*	103–140	106.0 *(10.8)*	91–133	<.001

	**Longitudinal Sample**	119.5 *(9.8)*	103–140	104.9 *(9.9)*	94–125	<.001

	***p*-value**	.40			.74		

**MABC-2 Total Scaled Score**	**Full Sample**	10.7 *(2.3)*	7–16	7.9 *(3.6)*	2–17	<.001

	**Longitudinal Sample**	11.0 *(2.7)*	7–16	8.6 *(3.7)*	4–17	.07

	***p*-value**	.75			.56		

*Note*. TD: Typical Development; DLD: Developmental Language Disorder; SPELT-P2/SPELT-3: Structured Photographic Expressive Language Test-Preschool 2 or Structured Photographic Expressive Language Test-Preschool 3; BBTOP: Bankson-Bernthal Test of Phonology; Nonverbal intelligence in the full sample was calculated using standard scores from the Columbia Mental Maturity Scale-3 (*n* = 49) and the Primary Test of Nonverbal Intelligence (*n* = 6); nonverbal intelligence in the longitudinal sample was calculated using standard scores from the Columbia Mental Maturity Scale-3 (*n* = 28); MABC-2: Movement Assessment Battery for Children-2.

a TD vs DLD *p*-values

b Full Sample vs Longitudinal Sample *p*-values

**Table 2.** Novel gestures and object referents

Object	Gesture	Handshape	Path	Orientation
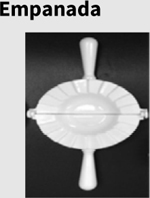	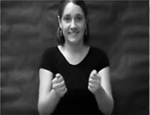	“A” in both hands	Both hands move to midline simultaneously along arced trajectory	Palms facing in towards midline

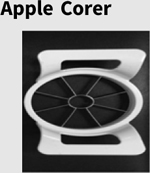	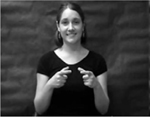	Bent “L” with thumb and index fingers in both hands	Both hands move downwards simultaneously	Palms facing in towards midline

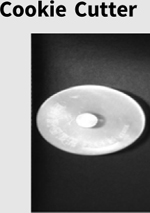	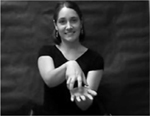	“C” in dominant hand; open “5” hand in nondominant hand	Dominant hand rotates; nondominant hand is stationary	Dominant hand palm is down; nondominant hand palm up

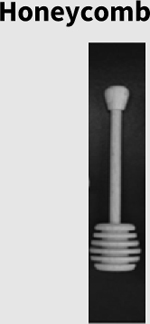	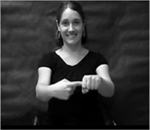	“1” in dominant hand inserted into closed “O” hand in nondominant hand	Both hands move away from body posterior to anterior	Dominant hand palm facing towards body; nondominant palm facing down

*Note*. Handshapes are described based on the ASL fingerspelling alphabet

**Table 3.** CELF-4 subtest group comparisons for the longitudinal cohort at time point three

		TD		DLD			
					
		Mean *(SD)*	Range	Mean *(SD)*	Range	*p*-value	η_p_^2^

**Time Point Three**	**Core Language Score**	108.8 *(12.5)*	90–126	89.0 *(12.3)*	67–114	<.001	0.30

	**Concepts & Following Directions**	10.9 *(1.4)*	7–13	8.3 *(2.9)*	2–13	.005	0.22

	**Word Structure**	12.0 *(2.5)*	6–15	7.6 *(3.1)*	2–13	<.001	0.28

	**Formulated Sentences**	13.0 *(2.5)*	9–17	9.6 *(2.1)*	6–14	.001	0.27

	**Recalling Sentences**	10.4 *(3.5)*	6–17	7.1 *(2.2)*	4–11	.006	0.20

*Note*. The DLD group sample size for the Concepts and Following Directions subtest, and subsequently the Core Language Score, is *n* = 12 because two participants did not complete the Concepts and Following Directions subtest.

**Table 4.** MABC-2 longitudinal cohort group means and standard deviations between time points

		TD		DLD			
					
		Mean *(SD)*	Range	Mean *(SD)*	Range	*p*-value	η_p_^2^

**Time Point One**	**Total Scaled Score**	11.0 *(2.7)*	7–16	8.6 *(3.7)*	4–17	.07	0.11

	**Manual Dexterity**	9.9 *(2.5)*	6–15	7.9 *(3.3)*	2–14	.10	0.09

	**Aiming & Catching**	10.4 *(2.8)*	5–16	10.4 *(2.8)*	6–16	1.00	0.00

	**Balance**	12.4 *(2.8)*	8–16	9.1 *(3.7)*	5–16	.02	0.17

**Time Point Three**	**Total Scaled Score**	10.4 *(2.3)*	7–16	8.6 *(3.6)*	3–16	.13	0.08

	**Manual Dexterity**	10.7 *(2.0)*	8–15	8.9 *(3.2)*	3–13	.09	0.10

	**Aiming & Catching**	8.9 *(3.2)*	4–15	8.6 *(4.3)*	5–18	.84	0.00

	**Balance**	11.3 *(2.4)*	7–16	8.8 *(3.0)*	3–16	.02	0.15

**Table 5.** Longitudinal cohort learning probes mean percent correct and standard deviations

	Comprehension			
	
	Time Point One		Time Point Three	
		
	Mean *(SD)*	Range	Mean *(SD)*	Range

**TD**	71% *(46%)*	—	93% *(26%)*	—

DLD	64% *(49%)*	—	100% *(0%)*	—

	Production			
	
	Time Point One		Time Point Three	
		
	Mean *(SD)*	Range	Mean *(SD)*	Range

**TD**	64% *(29%)*	0–100%	81% *(20%)*	33–100%

**DLD**	49% *(24%)*	0–100%	82% *(19%)*	33–100%

*Note*. There is no range reported for the comprehension probe because comprehension responses were indexed using a binary system of correct (1pt) or incorrect (0pts).

**Table 6.** Hierarchical analysis for the DLD group CELF-4 recalling sentences time point three language outcome (n=14)

Step & Predictor	Adj. R^2^	ΔR^2^	*F*	B	*SE* B	ß

**Step 1**	.04	.12	1.59			
		
(constant)				−0.72	6.27	
		
CMMS-3				0.08	0.06	.34

**Step 2**	.43	.40	5.92*			
		
(constant)				−9.35	5.61	
		
CMMS-3				0.06	0.05	.29
		
Shape				6.39	2.11	.64*

**Step 3**	.41	.03	4.01*			
		
(constant)				−12.62	7.09	
		
CMMS-3				0.07	0.05	.30
		
Shape				7.22	2.40	.72*
		
Path				1.23	1.58	.19

**Step 4**	.61	.18	6.02*			
		
(constant)				−17.08	6.07	
		
CMMS-3				0.09	0.04	.41*
		
Shape				6.12	2.01	.61*
		
Path				1.03	1.29	.16
		
Orientation				3.24	1.32	.45*

Note.

* *p* ≤ .05;

** *p* ≤ .01;

*** *p* ≤ .001.
